# Integration of qualitative and quantitative methods for land‐use‐change modeling in a deforestation frontier

**DOI:** 10.1111/cobi.13924

**Published:** 2022-06-17

**Authors:** Katherine Siegel, Aldo Farah Perez, Eva Kinnebrew, Megan Mills‐Novoa, José Ochoa, Elizabeth Shoffner

**Affiliations:** ^1^ Department of Environmental Science, Policy, & Management University of California, Berkeley Berkeley California USA; ^2^ Department of Ecology & Evolutionary Biology University of Colorado Boulder Boulder Colorado USA; ^3^ Department of Earth & the Environment Florida International University Miami Florida USA; ^4^ Rubenstein School of the Environment & Natural Resources and Gund Institute for Environment University of Vermont Burlington Vermont USA; ^5^ School of Geography & Development University of Arizona Tucson Arizona USA; ^6^ Energy and Resources Group University of California, Berkeley Berkeley California USA; ^7^ Geography Graduate Group University of California, Davis Davis California USA; ^8^ Department of Geography University of Washington Seattle Washington USA

**Keywords:** Amazon Basin, deforestation, discourse analysis, interdisciplinary, Jamanxim National Forest, land‐use change, protected area, remote sensing, análisis del discurso, área protegida, Bosque Nacional Jamanxim, cambio de uso de suelo, cuenca del Amazonas, deforestación, interdisciplinario, teledetección, 突变积累, 森林砍伐, 亚马逊流域, 话语分析, 遥感, 保护区, Jamanxim国家森林, 跨学科

## Abstract

Development and implementation of effective protected area management to reduce deforestation depend in part on identifying factors contributing to forest loss and areas at risk of conversion, but standard land‐use‐change modeling may not fully capture contextual factors that are not easily quantified. To better understand deforestation and agricultural expansion in Amazonian protected areas, we combined quantitative land‐use‐change modeling with qualitative discourse analysis in a case study of Brazil's Jamanxim National Forest. We modeled land‐use change from 2008 to 2018 and projected deforestation through 2028. We used variables identified in a review of studies that modeled land‐use change in the Amazon (e.g., variables related to agricultural suitability and economic accessibility) and from a critical discourse analysis that examined documents produced by different actors (e.g., government agencies and conservation nonprofit organizations) at various spatial scales. As measured by analysis of variance, McFadden's adjusted pseudo *R* ^2^, and quantity and allocation disagreement, we found that including variables in the model identified as important to deforestation dynamics through the qualitative discourse analysis (e.g., the proportion of unallocated public land, distance to proposed infrastructure developments, and density of recent fires) alongside more traditional variables (e.g., elevation, distance to roads, and protection status) improved the predictive ability of these models. Models that included discourse analysis variables and traditional variables explained up to 19.3% more of the observed variation in deforestation probability than a model that included only traditional variables and 4.1% more variation than a model with only discourse analysis variables. Our approach of integrating qualitative and quantitative methods in land‐use‐change modeling provides a framework for future interdisciplinary work in land‐use change.

## Resumen

El desarrollo y la implementación de la gestión efectiva de las áreas protegidas para reducir la deforestación dependen parcialmente de la identificación de los factores que contribuyen a la pérdida del bosque y de las áreas en riesgo de ser convertidas, pero el modelado estándar del cambio de uso de suelo puede no capturar completamente los factores contextuales que no se cuantifican fácilmente. Combinamos el modelado cuantitativo del cambio de uso de suelo con el análisis cualitativo del discurso en un estudio de caso del Bosque Nacional Jamanxim de Brasil para entender de mejor manera la deforestación y la expansión agrícola en las áreas protegidas del Amazonas. Modelamos el cambio de uso de suelo entre 2008 y 2018 y lo proyectamos hasta 2028. Usamos las variables identificadas en una revisión de estudios que modelaron el cambio de uso de suelo en el Amazonas (p. ej.: variables relacionadas con la idoneidad agrícola y la accesibilidad económica) y en el análisis crítico del discurso que examinó documentos producidos por diferentes actores (p. ej.: agencias gubernamentales y organizaciones sin fines de lucro para la conservación) a varias escalas espaciales. Conforme a las medidas del análisis de varianza, la pseudo‐R2 ajustada de McFadden y el desacuerdo en la cantidad y la asignación, descubrimos que la inclusión dentro del modelo de las variables identificadas como importantes para las dinámicas de deforestación mediante el análisis cualitativo del discurso (p. ej.: la proporción de terrenos públicos sin asignar, la distancia hacia los desarrollos propuestos de infraestructura y la densidad de incendios recientes) junto con variables más tradicionales (p. ej.: elevación, distancia a las carreteras y estado de protección) mejoró la habilidad predictiva de dichos modelos. Los modelos que incluyeron la mezcla de variables explicaron hasta 19.3% más de la variación observada en la probabilidad de deforestación que un modelo que solamente incluyó las variables tradicionales y 4.1% más variación que un modelo con las variables del análisis del discurso. Nuestra estrategia de integrar los métodos cualitativos y cuantitativos dentro del modelado del cambio de uso de suelo proporciona un marco para futuros trabajos interdisciplinarios sobre el cambio de uso de suelo.

## 整合定性和定量方法进行森林砍伐边界的土地利用变化建模

【摘要】制定和实施有效的保护区管理以减少森林砍伐, 部分取决于能否确定导致森林丧失的因素和面临土地转换风险的地区, 但标准的土地利用变化建模可能无法充分反映难以量化的背景因素。为了更好地了解亚马逊保护区的森林砍伐和农业扩张情况, 我们将定量的土地利用变化模型与定性的话语分析相结合, 对巴西Jamanxim国家森林进行了案例研究。我们模拟了2008‐2018年的土地利用变化情况, 并预测了到2028年的森林砍伐情况。本研究使用的变量来自一项对亚马逊地区土地利用变化建模研究的综述(如与农业适宜性和经济可及性相关的变量), 以及一项检验不同行为者(如政府机构和非营利性保护组织)在不同空间尺度上制定的文件的话语分析。通过方差分析、McFadden调整后的伪*R* ^2^, 以及数量分歧与分配分歧的计算, 我们发现, 在模型中加入通过定性话语分析确定的森林砍伐动态的重要变量(如未分配的公共土地比例、与计划修建的基础设施的距离、近期火灾密度)以及更传统的变量(如海拔、与道路的距离、保护状态)可以提高这些模型的预测能力。包含话语分析变量和传统变量的模型与只包含传统变量的模型相比, 可解释的森林砍伐可能性的观测变异高19.3%, 而与只包含话语分析变量的模型相比高4.1%。我们在土地利用变化建模中定性和定量相结合的方法可以为未来土地利用变化的跨学科工作提供框架。**【翻译:胡怡思;审校:聂永刚】**

## INTRODUCTION

Tropical forests face rapid and widespread conversion to anthropogenic land uses, with consequences for biodiversity, traditional land tenure and livelihoods, hydrology, and carbon emissions (Baragwanath & Bayi, 2020; Hansen et al., 2008; Malhi et al., 2008). In response, governments have pledged to set aside large amounts of remaining tropical forest in protected areas (PAs). PAs cover 15% of the world's land area and over 29% of tropical forests (Morales‐Hidalgo et al., 2015; UNEP‐WCMC & IUCN, 2020). Although PAs generally have reduced rates of deforestation within their boundaries (Andam et al., 2008; Geldmann et al., 2013), forest loss continues and varies with management objectives, location, land‐cover type, enforcement levels, and other factors (Geldmann et al., 2019; Leberger et al., 2020).

The Amazon rain forest represents a globally significant biodiversity hotspot that influences global carbon and regional hydrological cycles (Arima et al., 2014; Myers et al., 2000; Strand et al., 2018). The Amazon Basin covers 6 million km^2^ across 9 countries, including over 350 conservation or sustainable resource management areas (CAMP, 2018). Large swaths of forest have been converted to anthropogenic land uses: since 1970, forest cover declined by ∼15% (loss of 900,000 km^2^) (Amigo, 2020; Fearnside, 2005). Despite their status, protected forests throughout the region experience timber harvesting, small‐ and industrial‐scale livestock and agricultural expansion, infrastructure development, increases in human settlements, and mineral and fossil fuel extraction (Asner & Tupayachi, 2017; Viteri‐Salazar & Toledo, 2020). For example, PAs in the Brazilian Amazon lost 0.05–0.1% of their forest cover annually from 2002 to 2016 (Cabral et al., 2018), and Brazilian PAs near roads and navigable rivers lost 10.9% of their forest cover from 2000 to 2006 (Barber et al., 2014). While overall deforestation rates in the Brazilian Amazon decreased after 2004, they increased recently (INPE, 2020). President Jair Bolsonaro's administration, beginning in 2019, intensified land‐use pressures by undermining environmental agencies, eliminating regulations, reducing law enforcement, and promoting mining and agricultural incursions in Indigenous territories (Coelho‐Junior et al., 2022; Ferrante & Fearnside, 2019; Gomes Barbosa et al., 2021).

Deforestation in Amazonian PAs is part of complex environmental, political, and socioeconomic changes that vary locally (Jusys, 2016; Rosa, Purves, et al., 2014). Developing policies and management that conserve protected forests requires identifying the factors related to deforestation in specific locations (Ravikumar et al., 2017). Land‐use‐change modeling improves understanding of deforestation dynamics. Standard land‐use‐change models relate observed changes in land use or land cover to spatial, quantitative data on factors related to land‐use transitions (Verburg et al., 2004). These models identify drivers of deforestation (e.g., expanded cash crop cultivation, migration [Meyfroidt, 2015]), predict areas at risk of conversion, simulate impacts of different policy interventions, and explore future scenarios (Plantinga, 2021). They can inform debates about the drivers of and solutions to PA deforestation and are thus tools for land‐use planning, conservation prioritization, and management and policy interventions.

Despite the power of land‐use‐change models to explain and predict deforestation (Etter et al., 2006; Soares‐Filho et al., 2004), they do not always fully capture forest‐loss discourses. These discourses, which vary across spatial and temporal scales, reflect how actors understand deforestation and thus influence proposed policy interventions, shaping future land‐use change (Ravikumar et al., 2017). Integrating qualitative discourse analysis with land‐use‐change modeling requires synthesis of qualitative and quantitative methods and data (Kinnebrew et al., 2020). Many land‐use‐change models use quantitative explanatory variables previously related to the probability of deforestation, including distances to roads and cities, cropland suitability, and topography (Barber et al., 2014; Rosa, Ahmed, et al., 2014; Soares‐Filho et al., 2005). Although these variables undeniably influence land‐use‐change dynamics, they do not fully reflect discourses on factors contributing to deforestation in specific locations, such as land speculation and tenure, enforcement capacity, governance, and migration (Killeen et al., 2008; Martins et al., 2017).

Story and simulation (SAS) methods integrate qualitative data in quantitative models, drawing on written documents, key informant interviews, and stakeholder workshops (Alcamo, 2008; Vacquié et al., 2015) to model socioenvironmental scenarios. However, SAS methods have largely been applied to large‐scale scenario development to explore multiple future pathways. We applied a similarly integrative framework to spatial land‐use modeling with a focus on understanding current socioenvironmental dynamics.

We used mixed methods to integrate qualitative discourse analysis with quantitative land‐use‐change modeling to analyze the factors related to deforestation and project future deforestation in an Amazonian PA, Brazil's Jamanxim National Forest. We used explanatory environmental, geographic, social, and management variables from previous land‐use‐change models for the region and variables identified through a qualitative discourse analysis that identified the narratives created and circulated by national, state, and local actors to explain and address deforestation. This method facilitates understanding of different factors’ roles in contributing to or reducing the probability of deforestation (Kinnebrew et al., 2020; Ravikumar et al., 2017). To demonstrate the value of our approach, we quantified how the inclusion of discourse analysis variables changed which variables were important, model performance, and spatial patterns of predicted future deforestation.

## METHODS

### Study site

**FIGURE 1:**
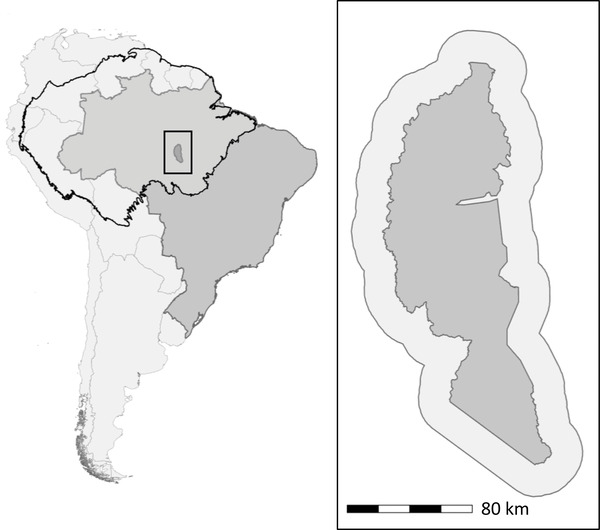
Study site in (a) South America (black outline, Amazon Basin; dark gray, Brazil) and (b) Jamanxim National Forest (dark gray) with a 20‐km area around it in lighter gray

We selected Brazil's Jamanxim National Forest (hereafter Jamanxim) as a case study because it has experienced relatively high levels of deforestation (Cabral et al., 2018). Jamanxim is in Pará state, has an area of 13,015 km^2^, and was established in 2006 to limit deforestation associated with construction of highway BR‐163 through the Amazon. As a national forest, Jamanxim is managed for sustainable use, including watershed protection and sustainable logging and silviculture, although no logging concessions have been granted (Rylands & Brandon, 2005). Following its establishment, the probability of deforestation inside Jamanxim decreased (Soares‐Filho et al., 2010), but it has since experienced significant forest loss through logging and land clearing for ranching and agriculture. In the absence of clear, well‐enforced land tenure, large‐ and small‐scale farmers clear the forest to secure land claims and establish farms and ranches (Campbell, 2015). Large‐scale landowners use deforestation as a form of land speculation: by clearing land, they increase its value (Miranda et al., 2019; Torres, 2012). Jamanxim has also faced legal threats. A 2008 bill proposed to degazette Jamanxim to resolve competing land claims in favor of farmers and ranchers (de Marques & Peres, 2014), and in 2012, Brazil's president temporarily reduced its size to allow for construction of a hydropower dam (Fearnside, 2016). We modeled land‐use change in Jamanxim and in a 20‐km area around the forest to capture land‐use dynamics just outside the management area (Figure 1). Because of the abrupt changes in the policy and politics of forest conservation of the Bolsonaro administration in 2019, we restricted our study period for model validation to 2008 to 2018.

### Remote sensing

We generated 2008 and 2018 land‐cover maps with supervised classifications of cloud‐free composites of Landsat 5 (TM), Landsat 7 (ETM+), and Landsat 8 (OLI) 30‐m Surface Reflectance data sets with random forests. We used dry‐season (May 15 to October 15) data and masked clouds with the CFMask algorithm (Foga et al., 2017). To better distinguish among land‐cover types with seasonal variation, we integrated elevation data (SRTM Digital Elevation Data at 30 m), enhanced vegetation index (EVI), and the difference in seasonal EVI between wet and dry seasons (Liu & Huete, 1995) (methods described in Appendix S1).

We developed our training data set with manual classifications of land cover in Geosurvey, which integrates 1‐m‐resolution imagery from Bing Aerial, Google Hybrid, and Matchbox (QED, 2019). To classify land‐cover types, we drew polygons for agriculture and pastures, forest, bare soil, built areas, wetlands, and water in 1000 randomly selected 250 × 250 m windows. Each window could contain multiple polygons with different land‐cover types. In Google Earth Engine, we performed a supervised classification with random forest algorithms with our spectral imagery, training polygons, and additional elevation and EVI seasonal difference data (Belgiu & Drăguţ, 2016; Breiman, 2001). We used a 10,000‐fold cross validation to validate classification accuracy in the dismo package in R (Hijmans et al., 2017). Due to inaccurate classifications for nonforest land‐cover types, we classified up to 100 more 250 × 250 m polygons in the agriculture, bare soil, built, water, and wetland land covers. Including these additional polygons improved classification accuracy, although uncertainty in the validation methods remained, given our use of manual imagery classification.

### Qualitative discourse analysis

We used qualitative discourse analysis methods to analyze the discourse around the drivers and mediators of deforestation in Jamanxim. We sampled documents in English and Portuguese that discussed deforestation in the PA (sampling and coding details in Appendix S2). We sampled 4 types of documents at the national, state, and park scales: management (e.g., park management plans), policy (e.g., laws and decrees related to PAs and forest management), gray literature (e.g., government agency and nongovernmental organization [NGO] reports), and advocacy (e.g., articles written by NGOs to promote campaigns or support certain arguments). We used snowball sampling methods to compile policy and management documents related to Jamanxim from the Brazilian government's legislative database (Federal Government of Brazil, 2019). Search terms were “*Jamanxim National Forest*” or “*Floresta Nacional* *do* *Jamanxim*” or *Pará*, plus *deforestation*, *desmatamento*, and “*agricultural expansion*” or closely related terms. We sampled gray literature and advocacy documents by identifying all NGOs working in the area and locating their online publications. We sampled to the point of saturation, including documents based on relevance and repetition. Our final sample consisted of 61 documents (5 management, 12 policy, 23 gray literature, and 21 advocacy [Appendix S3]).

We coded these documents in NVivo 12 (QSR International, 2019) based on predetermined (initial) and emergent themes (Appendices S5 & S6). We developed the list of initial themes based on a literature review of variables used in Amazonian land‐use‐change modeling (Appendix S4) and a word count in NVivo 12 of all sampled documents. Emergent themes arose during the process of coding a subsample of the documents. When a theme appeared in >3 documents, we considered it an emergent theme and included it in our coding process for the remaining documents (Appendix S6).

### Land‐use‐change models

To assess the role of the different types of variables in explaining deforestation trends, we constructed 4 logistic regression models to explain and predict forest conversion to agriculture (Moulds et al., 2015; Rosa, Ahmed, et al., 2014). First, we built a model with variables derived from the land‐use‐change literature (LUC model). Our second model consisted of the variables identified through qualitative discourse analysis (see “Variable selection” section). The third model included all the variables from the LUC model and DA Model (LUC and DA model), and the fourth model (refined LUC and DA model) included the variables that were statistically significant in the LUC model and variables determined to be highly important through the qualitative discourse analysis. For each model, we checked for correlations between the continuous variables and removed highly correlated variables (Pearson's correlation coefficient >0.66, *p* < 0.05) prior to model runs. When choosing between 2 highly correlated variables, we retained variables based on the year of the data (with a preference for data from as close to the beginning of our study period as possible), data quality, and hypothesized strength of the relationship with forest conversion.

To control for spatial autocorrelation, we extracted cell values for forested pixels along a 300‐m grid to avoid including values from adjacent pixels; included the *x* and *y* coordinates of the observations as model covariates, unless they were highly correlated with other explanatory variables (Schleicher et al., 2017); and included as an explanatory variable the proportion of surrounding pixels that were nonforest land‐cover classes (Gibbons & Overman, 2012).

#### Variable selection

For the LUC model, we used a suite of variables that are frequently used in land‐use‐change models in the Amazon or that have been linked to deforestation risk in the region (Appendix S4): distance to the nearest road, river, and city (proxies for accessibility and distance to markets); population density; elevation, slope, aspect, soil moisture, and precipitation (proxies for agricultural suitability); crop suitability; management status; poverty rate; and distance to mining concessions.

For the DA model, we used variables related to the dominant narratives around the drivers of and solutions to deforestation in Jamanxim, as identified through our qualitative discourse analysis. We identified themes and determined quantitative spatial proxies for each theme for use in the models based on literature reviews and the best available data (Alcamo, 2008; Mallampalli et al., 2016). For example, an important theme was land grabbing (*grilagem*), which often occurs on publicly owned land that has not yet been granted an official use (e.g., logging concessions, PA designation) (Campbell, 2015; Torres, 2012). Because there is no database of the locations of illegal clearing of public land, we used the percentage of unallocated public land in the municipality as a spatial, quantitative proxy for the conditions that facilitate the process of land grabbing. The number of themes identified was not predetermined, but rather emerged through the coding process. After removing factors we could not translate into spatial quantitative proxies, we had 10 variables: distances to existing agriculture, fires, proposed infrastructure developments (railroads and dams), and unauthorized mines; density of past fires; percent unallocated public land in the municipality; areas proposed for PA downgrading, downsizing, and degazettement (PADDD); presence of agricultural reform settlements; and head of cattle per square kilometer (Table 1). We removed distance to proposed dams because it was highly correlated with distance to unauthorized mining sites.

**TABLE 1:** Themes identified in the qualitative discourse analysis of management, policy, gray literature, and advocacy documents related to deforestation and agricultural expansion in Jamanxim National Forest, the state of Pará, and Brazil and their corresponding quantitative spatial proxies

Theme	Proxy
Agriculture	distance to existing agricultural land (m)
Fires	distance to fires (2007–2018) (m); fire density (2007–2018) (per km^2^)
Unauthorized mining	distance to unauthorized mining sites (m)
Legal threats to protected areas	presence of PADDD proposals
Land grabbing	proportion of unallocated public land
Ranching	head of cattle per km^2^
Infrastructure development	distance to proposed railroads (m); distance to proposed dams (m)
Land tenure, settlements	presence of agricultural reform settlements

Abbreviation: protected area downgrading, downsizing, and degazettement.

The LUC and DA model included all variables from the LUC model and the DA model except for variables that were highly correlated: we dropped head of cattle per square kilometer because it was highly correlated with population density (Pearson's correlation coefficient = –0.99, *p* < 0.05). The refined LUC and DA model included 9 variables from the LUC model (slope; elevation; distance to roads, cities, and mining concessions; crop suitability; soil moisture; protection status; percentage of surrounding nonforest pixels) and 6 variables from the DA model (distance to existing agriculture, fires, and proposed railroads, PADDD status, fire density, and agricultural reform settlements) (Appendices S5 & S6).

In all models, we included whether the forested point was in Jamanxim, within a 10‐km area (hereafter buffer) around the national forest, or within a 20‐km buffer. We included the buffer because the presence of PAs frequently affects land‐use pressure in the surrounding area (Ewers & Rodrigues, 2008) and we wanted to capture these dynamics and the processes of agricultural encroachment from the buffer into the PA. Ten‐ and 20‐km buffers are relevant to land‐use dynamics in PAs (Tesfaw et al., 2018).

#### Data compilation

We compiled quantitative data on the variables from local, national, regional, and global sources (Table 2). Where possible, we matched the temporal scale of the data to the time frame of our study (2008–2018). We converted all data to rasters with resolutions matching our land‐cover‐change maps (30 × 30 m). We standardized the spatial data in QGIS and R with the raster, sf, and lwgeom packages (Hijmans, 2019; Pebesma, 2019, 2018; QGIS Development Team, 2019).

**TABLE 2:** Variables and spatial proxies used in models of forest conversion to agriculture in Jamanxim National Forest from 2008 to 2018

Variable	Type of variable and justification for inclusion	Original data resolution or format (processing method, where applicable)	Year of original data collection or generation	Source
	Standard land‐use change (LUC) variables			
Elevation (m), slope (°), and aspect (°)	measure of suitability for alternative land uses	1 arc second	2000	Farr et al., 2007
Distance to nearest road (m)	measure of accessibility	vector (Euclidean distance)	2019	Open Street Map, 2019
Distance to nearest river (m)	measure of accessibility	vector (Euclidean distance)	1992	DIVA‐GIS, 2019
Distance to nearest city (m)	measure of accessibility	vector (Euclidean distance)	2010	IBGE, 2010
Mean population density (per km^2^)	measure of pressure for land conversion	vector at municipality scale	2010	IBGE, 2010
Precipitation (mm)*	proxy for agricultural suitability	0.05 arc degrees	2008–2018 (mean daily value)	Funk et al., 2015
Surface soil moisture (mm)	proxy for agricultural suitability	0.25 arc degrees, 2016–2018 average	2016–2018 (mean daily value)	O'Neill et al., 2016
Crop suitability (metric integrating climate, topography, and soil properties)	index of agricultural suitability	30 arc seconds	modeled for 1981–2010	Zabel et al., 2014
Poverty rate*	wealthier land owners may deforest more	vector at municipality scale	2000	IBGE, 2003
Distance to mining concessions (m)	measure of accessibility and localized human land use	vector (Euclidean distance)	2018	ANM, 2019
Protection status	presence of protected areas affects land use trends	vector	2018	IUCN & UNEP‐WCMC, 2019
Proportion of neighboring cells with a different land cover type	accounts for neighborhood effects and expansion of alternative land‐cover types	30 m	2008	derived from land‐cover maps
	Discourse analysis (DA) variables			
Distance to existing agricultural land (m)	proximity to agriculture increases probability of deforestation	30 m	2008	derived from land‐cover maps
Distance to fires (2007–2018) (m)	fire is part of land conversion process	vector (Euclidean distance)	2007–2018	INPE, 2019
Fire density (2007–2018) (per km^2^)	fire is part of land conversion process	vector	2007–2018	INPE, 2019
Distance to unauthorized mining sites (m)	unauthorized mining contributes to forest conversion and is part of the land conversion process	vector (Euclidean distance)	2018	RAISG, 2018
Presence of PADDD proposals	areas with proposed or implemented protected area down grading, downsizing, and de‐gazettement (PADDD) are at risk of deforestation		through 2018	Conservation International & World Wildlife Fund, 2019
Proportion of unallocated public land	lack of an officially designated owner or land use type incentivizes forest conversion	vector at municipality scale	as of 2014–2018	Imaflora & GeoLab, 2018
Head of cattle per km^2^ *	ranching is a cause of deforestation	vector at municipality scale	2017	IBGE, 2017
Distance to proposed railroads (m)	infrastructure development causes deforestation	vector (Euclidean distance)	through 2018	Ministério da Infraestrutura, 2019
Distance to proposed dams (m)*	infrastructure development causes deforestation	vector (Euclidean distance)	through 2018	ANEEL, 2020
Presence of agricultural reform settlements	secure land tenure is a factor that reduces deforestation	vector	through 2018	INCRA, 2021

* Excluded from models due to correlation with another variable.

#### Model performance

To compare model performance, we used analysis‐of‐variance (ANOVA) comparisons of model fit and McFadden's adjusted pseudo *R* ^2^, which takes the number of explanatory variables into account (Hebbali, 2020).

To assess the different models’ ability to accurately predict forest conversion to agriculture, we used each of the 4 logistic regression models to generate a raster layer with the predicted probability of conversion to agriculture for each forested pixel in 2008. We then used Monte Carlo simulations to generate 1000 projected landscapes for 2018 for each model, based on the predicted probability maps. For all pixels with nonforest land‐cover types in 2008, we assumed no change in land cover from 2008 to 2018. We calculated the predicted deforestation rate for each model as the mean area of forest loss across these simulations. We used these simulations to generate predicted deforestation maps for 2018. Given the observed amount of forest conversion to agriculture from 2008 to 2018, we allocated forest loss to pixels that converted in the highest number of simulations for each model until we reached the predetermined forest loss area. This yielded 1 predicted landscape for each model. We compared these predicted landscapes with the observed 2018 landscape to calculate quantity and allocation disagreement, where quantity disagreement was the difference in the proportion of pixels in each land‐cover category in the predicted and observed maps, and allocation disagreement was the amount of difference between the predicted and observed maps caused by spatial mismatch in the location of the pixels in each land‐cover class (Pontius & Millones, 2011). We calculated both measures of disagreement with the package diffeR (Pontius Jr. & Santacruz, 2019).

#### Predicted future deforestation

To assess the different models’ predicted spatial trends of deforestation, we used each model to generate a predicted probability of forest loss in 2028 by applying the models to the 2018 landscape. We used the previously described Monte Carlo method to simulate 1000 landscapes for 2028 for each model, assuming static relationships between the explanatory variables and deforestation risk over time. We updated the calculations of distance to agriculture and the proportion of nonforest neighboring pixels based on the observed 2018 land‐cover map. We used the observed 2008–2018 deforestation rate to generate predicted land‐cover maps for 2028. With a set area of conversion, we allocated forest loss to the pixels that converted in the highest number of simulations for each model until we reached the predetermined area of forest loss. This yielded 1 predicted landscape for each model.

#### Landscape connectivity

To quantify the different spatial distributions of the models’ predicted deforestation, we calculated metrics of landscape connectivity and fragmentation for all observed and projected landscapes in the R package landscapemetrics (Hesselbarth et al., 2019). Our landscape metrics analyses focused on the class level, combining all forested patches, and used 8‐cell neighbor methods (Rosa et al., 2017). We calculated connectivity and fragmentation metrics related to overall forest area (class area), fragmentation of contiguous forest area (patch area, number of patches, landscape division index), amount of core forest area (core area index, core area as percentage of landscape, total core area), and patch complexity and edge effects (fractal dimension index, perimeter–area fractal dimension, perimeter:area ratio, total edge).

## RESULTS

### Observed land use and land‐cover change

**FIGURE 2:**
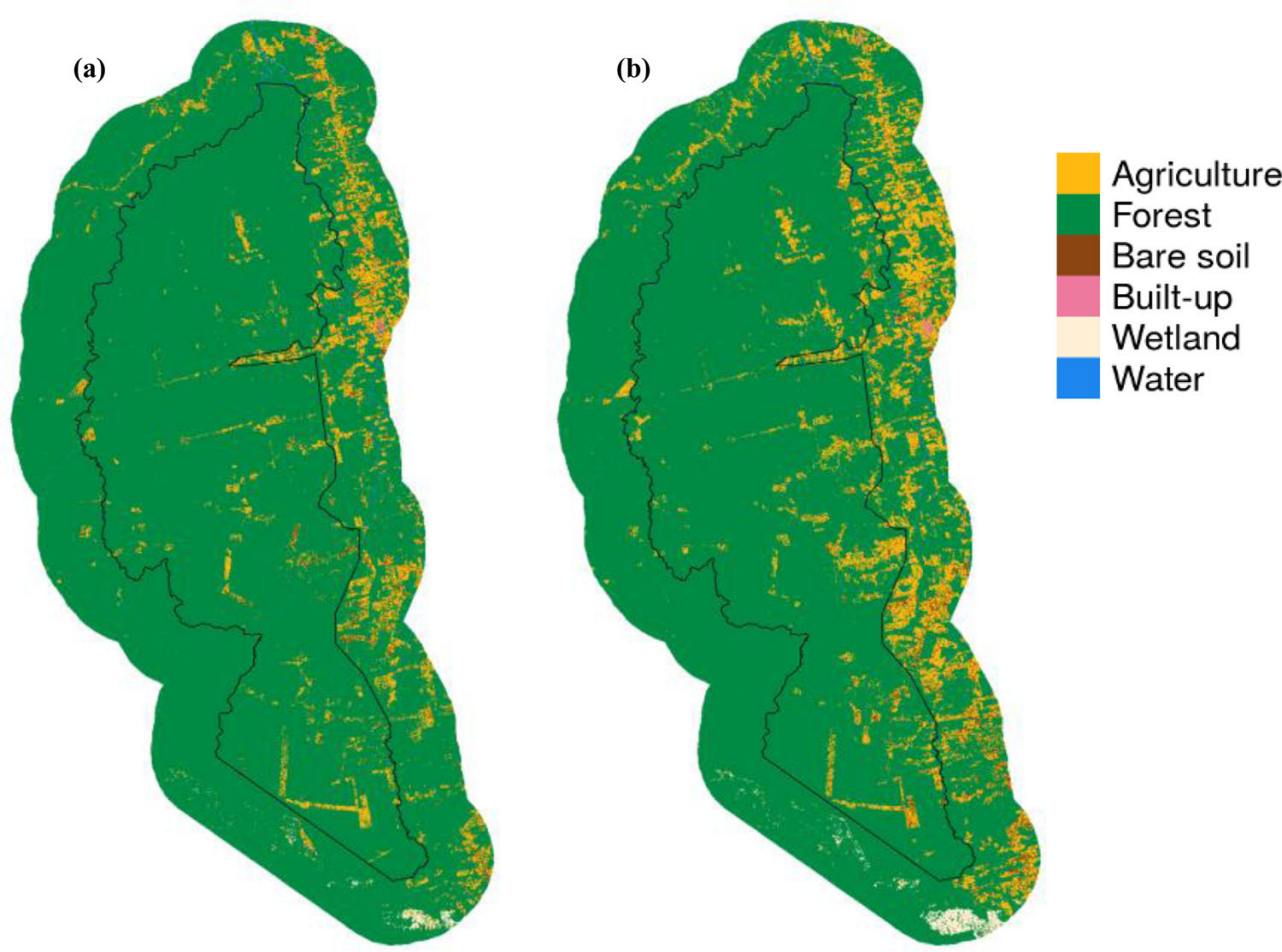
Land cover in (a) 2008 and (b) 2018 in Jamanxim National Forest and a 20‐km area surrounding the national forest (black line, national forest boundary)

Our remote sensing yielded high accuracy rates: average producer's and user's accuracy values were 94% and 91%, respectively (Appendix S7). We observed a 4.6% decline in forest cover from 2008 to 2018 in Jamanxim (1164.81 km^2^ of forest lost) (Figure 2). Each forested pixel in Jamanxim had a 5.0% probability of converting to agriculture from 2008 to 2018 (Appendix S8). Other land‐cover conversions with relatively high probabilities of occurrence were agriculture to forest (18.1%, 293.26 km^2^), agriculture to bare soil (10.7%, 172.87 km^2^), and bare soil to agriculture (68.0%, 196.43 km^2^). Mean area of forest patches decreased from 2008 to 2018, whereas the number of forested patches increased. The mean of the core area index (percentage of each patch that is core area) also increased, whereas measures of patch complexity did not change meaningfully. The total area of core forest decreased and the length of the total edges increased (Table 3).

**TABLE 3:** Landscape metric values for the observed landscape of Jamanxim National Forest in 2008 and 2018 and projected landscapes in 2028 under four different models of forest conversion to agriculture

	2008	2018	2028			
Metric	observed (SD)	observed (SD)	Land‐use change (LUC) model (SD)	Discourse analysis (DA) model (SD)	LUC and DA model (SD)	Refined LUC and DA model (SD)
Mean patch area (ha)	187.54 (15,310.33)	134.32 (12,770.25)	286.67 (18,402.11)	166.44 (14,028.57)	377.12 (21,129.24)	375.57 (21,077.05)
Number of patches	6696	9111	4151	7151	3156	3169
Class area (ha)	1,255,766.43	1,223,813.04	1,189,969.76	1,190,247.12	1,190,188.47	1,190,180.10
Mean of core area index	1.17 (5.24)	1.77 (6.79)	4.26 (11.22)	1.65 (6.78)	5.04 (12.12)	4.95 (12.15)
Mean of core areas of all patches (ha)	183.76 (15,031.31)	131.19 (12,512.43)	278.46 (17,918.71)	162.45 (13,724.26)	368.98 (20,710.56)	367.47 (20,659.93)
Core area as percentage of landscape	94.53	91.83	88.91	89.36	89.57	89.57
Landscape division index	0.07	0.12	0.17	0.17	0.17	0.17
Mean fractal dimension index	1.03 (0.05)	1.04 (0.05)	1.04 (0.05)	1.03 (0.05)	1.04 (0.05)	1.04 (0.05)
Perimeter‐area fractal dimension	1.49	1.45	1.38	1.46	1.35	1.35
Mean perimeter:area ratio	0.11 (0.03)	0.11 (0.03)	0.10 (0.03)	0.11 (0.03)	0.09 (0.03)	0.09 (0.03)
Percentage of landscape forested	96.47	94.02	91.53	91.55	91.55	91.55
Total core area (ha)	1,230,444	1,195,300	1,155,876	1,161,696	1,164,510	1,164,509
Total edge (m)	12,419,796	14,149,824	12,746,011	11,148,707	8,751,671	8,735,984

### Relationships between explanatory variables and deforestation probability

In the LUC model, elevation, slope, soil moisture, population density, and distances to roads and mining concessions had negative relationships with the probability of forest conversion to agriculture (Table 4). Distance to cities, crop suitability, percentage of nonforest neighboring pixels, and location in the buffer rather than the national forest were all associated with a higher probability of forest conversion. In this model, forested points at higher elevations and on steeper slopes with higher soil moisture and located further from roads and mining concessions were less likely to convert to agriculture, whereas forested points located outside the PA boundary, further from cities, with greater agricultural suitability and more adjacent nonforest areas were more likely to convert. Aspect and distance to rivers did not have significant relationships with deforestation probability in this model.

**TABLE 4:** Coefficient estimates (SD) for all models of forest conversion to agriculture in Jamanxim National Forest from 2008 to 2018

Variable	Land‐use change (LUC) model	Discourse‐analysis (DA) model	LUC and DA model	Refined LUC and DA model
Intercept	11.358^***^ (0.559)	–0.795^***^ (0.044)	8.504^***^ (0.924)	6.723^***^ (0.825)
Aspect (°)	1.390 × 10^–4^ (0.000)		2.063 × 10^–4^ (0.000)	
Slope (°)	–0.048^***^ (0.003)		–0.055^***^ (0.003)	–0.055^***^ (0.003)
Elevation (m)	–0.003^***^ (0.000)		–0.002^***^ (0.000)	–0.002^***^ (0.000)
Distance to roads (m)	–0.009^***^ (0.000)		–0.001^**^ (0.000)	–0.001^**^ (0.000)
Distance to rivers (m)	–2.703 × 10^–7^ (0.000)		1.601 × 10^–5***^ (0.000)	
Distance to mining concessions (m)	–0.003^***^ (0.000)		–2.901 × 10^–4^ (0.000)	–8.972 × 10^–5^ (0.000)
Distance to cities (m)	1.259 × 10^–6***^ (0.000)		–5.114 × 10^–6***^ (0.000)	–3.436 × 10^–6***^ (0.000)
Crop suitability	0.020^***^ (0.004)		–0.009^*^ (0.004)	–0.007 (0.004)
Population density (per km^2^)	–0.872^***^ (0.046)		0.452 (0.639)	
Soil moisture (mm)	–0.706^***^ (0.028)		–0.423^***^ (0.042)	–0.334^***^ (0.041)
Proportion of nonforest neighboring pixels	0.059^***^ (0.001)		0.041^***^ (0.001)	0.041^***^ (0.001)
10‐km buffer	0.796^***^ (0.026)		0.477^***^ (0.051)	0.427^***^ (0.050)
20‐km buffer	0.799^***^ (0.029)		0.344^***^ (0.057)	0.307^***^ (0.057)
Protected area downgrading, downsizing, and degazettement proposal		–0.079^**^ (0.026)	0.274^***^ (0.046)	0.250^***^ (0.045)
Proportion of unallocated public land		–0.118 (0.162)	–3.718 (2.265)	–1.524^***^ (0.217)
Distance to unauthorized mining sites (m)		–2.386 × 10^–6***^ (0.000)	5.729 × 10^–6***^ (0.000)	
Distance to existing agriculture (m)		–0.002^***^ (0.000)	–0.001^***^ (0.000)	–0.001^***^ (0.000)
Distance to fires (m)		–0.002^***^ (0.000)	–0.002^***^ (0.000)	–0.002^***^ (0.000)
Fire density (per km^2^)		0.047^***^ (0.001)	0.049^***^ (0.001)	0.049^***^ (0.001)
Distance to proposed railroads (m)		–1.183 × 10^–5***^ (0.000)	2.006 × 10^–6^ (0.000)	–1.871 × 10^–6^ (0.000)
Presence of agricultural reform settlements		–0.121^**^ (0.042)	0.125^**^ (0.046)	0.105^*^ (0.045)

^*^ *p* < 0.05; ^**^ *p* < 0.01; ^**^ *p* < 0.001.

The DA model indicated that the distance to unauthorized mining sites, existing agriculture, fires, and proposed railroads had negative relationships with the probability of forest conversion to agriculture, as did location in an area proposed for PADDD and the presence of agricultural reform settlements, the proxy variable for land tenure (Table 4). Fire density related positively to forest conversion probability, whereas the proportion of unallocated public land (proxy for land grabbing) had no significant relationship with deforestation. Forested points located farther from areas of extractive and agricultural activity and proposed infrastructure had a reduced conversion probability, as did points located in agricultural reform settlements or areas proposed for PADDD, whereas forests with greater fire density were more likely to convert.

In the LUC and DA and refined LUC and DA models, elevation, slope, soil moisture, and distance to roads, cities, existing agriculture, and fires all had negative relationships with the probability of forest conversion to agriculture (Table 4). In the LUC and DA model, crop suitability also had a significant, negative relationship with deforestation, whereas the same was true for the proportion of unallocated public land in the refined LUC and DA model. In both models, the proportion of nonforest neighboring pixels, fire density, and location in the buffer, areas proposed for PADDD, and agricultural reform settlements had positive relationships with deforestation probability. Forest points with a higher percentage of nonforested neighbors were more likely to convert to agriculture, as were sites located outside of the national forest's boundaries, sites that had been proposed for PADDD, and forests located in agricultural reform settlements. In the LUC and DA model, distance to rivers also had a positive relationship with deforestation, but this variable was not included in the refined LUC and DA model. The distances to the nearest mining concession and proposed railroad were insignificant in both models, as were aspect, population density, and the proportion of unallocated public land in the LUC and DA model and crop suitability in the refined LUC and DA model.

### Differences in model performance

All 4 models predicted higher deforestation rates from 2008 to 2018 than we observed. The LUC model predicted the lowest deforestation rate (1206.5 km^2^ [SD 0.9]), whereas the DA model predicted the highest level of deforestation (1286.9 km^2^ [0.9]). Based on ANOVA analysis, the refined LUC and DA model had the best model fit (*p* < 0.001). The LUC and DA model also outperformed the LUC model and the DA model (*p* < 0.001). The LUC and DA and refined LUC and DA models explained the greatest amount of variation in the observed forest conversion to agriculture from 2008 to 2018 (McFadden's adjusted pseudo *R* ^2^ values of 43.7% and 43.6%, respectively). The DA model explained more of the variation than the LUC model (39.5% vs. 24.4%). The 4 models had similar levels of quantity disagreement (0.016–0.017), and the LUC and DA and refined LUC and DA models had the lowest levels of allocation disagreement (0.057) (Table 5).

**TABLE 5:** Quantity and allocation disagreement for each of the 4 models of forest conversion to agriculture in Jamanxim National Forest from 2008 to 2018, based on comparisons of the observed 2018 land‐cover map with the predicted 2018 land‐cover maps generated with each model

Model	Quantity disagreement	Allocation disagreement
LUC	0.017	0.063
DA	0.016	0.059
LUC and DA	0.017	0.057
Refined LUC and DA	0.017	0.057

Abbreviations: DA, discourse analysis; LUC, land‐use change.

### Projected deforestation

Within the boundaries of the national forest from 2008 to 2018, the 4 models predicted 394.80–450.21 km^2^ of forest conversion to agriculture, compared with an observed loss of 319.53 km^2^. The LUC model predicted the lowest level of deforestation, and the DA model predicted the highest deforestation levels within Jamanxim's borders.

Although the observed number of forest patches increased from 2008 to 2018, all 4 models showed a decrease in the number of forest patches in 2028 relative to 2018 and an increase in the mean patch area. However, only the DA model predicted a decrease in mean patch area and increase in patch number relative to 2008. The LUC, LUC and DA, and refined LUC and DA models displayed an increase in the mean of the core area index from 2018 to 2028, whereas the DA model showed the opposite trend (Table 3).

**FIGURE 3:**
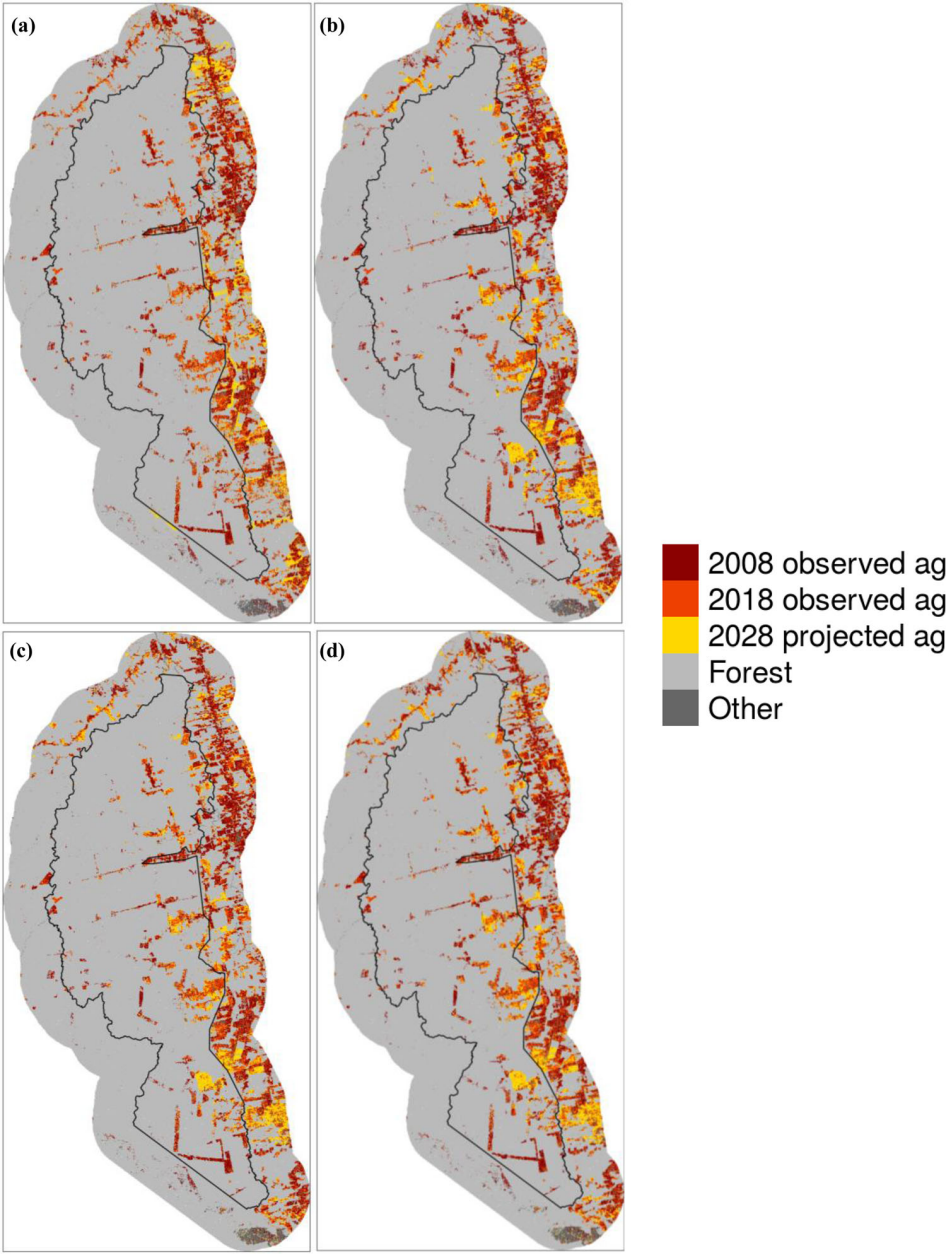
Forest conversion to agriculture observed from 2008 to 2018 and projected conversion to agriculture in and near Jamanxim National Forest based on the (a) land‐use change (LUC) model, (b) discourse analysis (DA) model, (c) LUC and DA model, and (d) refined LUC and DA model (ag, agriculture; yellow, projections based on the 4 different models; black line, national forest boundary)

The spatial distribution of predicted future deforestation varied between the models, but the 4 models’ predictions had some overlap: 352.7 km^2^, of which 75.6 km^2^ were within Jamanxim's boundaries (Figure 3). The areas where all 4 models predicted agricultural conversion by 2028 were mostly adjacent to areas with agriculture in 2008 and 2018, often forming thin perimeters around existing agriculture. There were also contiguous blocks of projected conversion around the BR‐163 highway and rivers in the eastern part of the buffer. Three of the 4 models predicted a large block of forest conversion in the southern part of Jamanxim.

## DISCUSSION

Our findings demonstrate the value of integrating qualitative and quantitative research methods for studying land‐use change. Our approach also contributes to larger scale and multifactor SAS scenario‐building efforts by outlining a discourse‐analysis‐based approach to land‐use‐change modeling. We adopted a similar integrative approach to SAS but created a probabilistic model of the future based on current dynamics. Our models that included variables from traditional land‐use‐change modeling approaches and discourse analysis had the best performance and predictive ability. Beyond providing a template for future efforts to combine qualitative and quantitative methods to understand land‐use change, our results indicated that the narratives about deforestation in PAs, which emerged across scales and actors, are important sources of information for understanding and predicting deforestation processes. When attempts to understand and predict deforestation omit information from these narratives, they may overlook significant factors related to forest loss and have reduced ability to predict locations at risk of deforestation.

Many of our results regarding the effects of individual variables on deforestation probability strengthen previous findings. For example, past analyses of deforestation in the Brazilian Amazon show that forested areas located closer to roads and previously deforested areas are more likely to lose forest cover, whereas PAs have lower rates of deforestation, although this varies among different PA categories (Nolte et al., 2013; Pfaff et al., 2015; Rosa, Purves, et al., 2014; Rosa et al., 2013). Similarly, our finding that areas at higher elevation and with steeper slopes were less likely to experience deforestation aligns with results from elsewhere in the Amazon (Muller et al., 2011), and our results linking fire activity to agricultural conversion aligned with observations that fire is a tool for land clearing (Escobar, 2019).

The relationship between the proportion of unallocated public land and deforestation probability did not match our expectations. There was notable discourse around land grabbing and illegal occupation of public land in the documents analyzed (e.g., Abdala, 2015; Araújo et al., 2017; PPCDAm & PPCerrado, 2016), and we expected to find less of this opportunistic land grabbing in areas where the tenure status of a greater percentage of public land had been resolved. However, we observed the opposite. Areas with greater proportions of unallocated public land experienced lower probabilities of deforestation. This may be the result of varying dynamics in the different municipalities included in the study area because our data on unallocated public land were at the municipality level. Our study area included parts of 3 municipalities: Novo Progresso, Itaituba, and Altamira. Altamira had at least a 5 times greater proportion of unallocated public land than the other municipalities, but it is possible that other land use trends or underlying agricultural suitability in Altamira or the other municipalities affected the role of land tenure in our models.

The DA model predicted the highest levels of forest conversion to agriculture, whereas the LUC model predicted the lowest deforestation rate. The mean projected deforestation rate for all models was greater than the observed deforestation rate from 2008 to 2018, highlighting the limitations of land‐use‐change models. The LUC model came closest to correctly estimating the area of forest converted to agriculture; the inclusion of the discourse‐analysis variables led to higher estimates of deforestation. This implies that an understanding of deforestation trends rooted solely in the discourses surrounding forest loss might lead to disproportionate or poorly targeted management. Although discourse analysis improved land‐use‐change modeling, management responses guided by discourses alone may not be effective.

### Spatial distribution of predicted deforestation

In 3 other regions of Brazil's Amazon, agricultural expansion increased forest fragmentation and the density of forest edges (Rosa et al., 2017). Although we observed a similar increase in fragmentation and forest edge density from 2008 to 2018, our models predicted a decrease in edge density from 2018 to 2028. We did not observe or predict notable changes in patch complexity over time, in contrast to expectations that forest fragmentation increases patch complexity (Wang et al., 2017). All models that included LUC variables predicted increased mean forest patch area and decreased number of forest patches from 2018 to 2028, indicating that these models projected conversion of small forest patches to agriculture. Notably, the DA model predicted an increase in mean patch area relative to 2018 (although still a decrease from 2008) and a concurrent decrease in the number of forested patches, contrary to the other 3 models. This implies that the inclusion of the LUC model variables (e.g., the proportion of nonforest neighboring pixels) that may capture “contagious” deforestation dynamics in which pixels surrounded by previously deforested land are more likely to experience forest loss (Robalino & Pfaff, 2012) drives this pattern.

The different variables used for each model drove differences in the spatial distribution of projected future deforestation. In particular, the models that included fire density (DA model, LUC and DA model, and refined LUC and DA model) predicted clusters of deforestation in the southeast portion of Jamanxim and the buffer. These areas had high fire densities from 2007 to 2018. Additional variables colocated with areas of predicted deforestation in those 3 models were the presence of agricultural reform settlements in the southeastern region of the buffer and PADDD proposals along the eastern border of Jamanxim.

Brazil's environmental policies and politics changed when President Bolsonaro took office in 2019 (Abessa et al., 2019; Escobar, 2020). More recent discourses around deforestation likely reflect this shift, and the relationships we observed between explanatory variables and deforestation probabilities likely have changed. Thus, our projections for forest conversion in 2028 should be interpreted with care and are most useful as a demonstration of the different predictions generated by standard land‐use‐change models as opposed to those that integrate discourses.

### Further integration of discourse analysis and land‐use‐change modeling

Incorporating discourse analysis into our method improved our models, but ideally, we would have even greater integration of variables identified in the discourse analysis into the models. The discourse analysis identified important themes that we were unable to include in the land‐use‐change models due to a lack of data for spatial, quantitative proxies. This included themes such as state capacity, commodity traceability, inclusion and participation in the management process, and enforcement capacity. We were also unable to include factors that lacked spatial variation, such as state‐level policies, because these factors’ values would be constant across the modeled landscape (although their implementation might not be uniform). These 2 categories of factors appeared frequently in the discourses around the drivers of deforestation in Jamanxim, Pará, and Brazil and likely influence deforestation dynamics. Our inability to translate these themes into quantitative, spatial proxies for inclusion in land‐use‐change modeling highlights a limitation of our method in particular and quantitative modeling in general; these approaches cannot accommodate important variables that are not easily measured and incorporated into a GIS framework. This demonstrates the need for qualitative analyses in addition to quantitative analyses when considering socioenvironmental change (Bennett et al., 2017). For other themes, the spatial, quantitative variables for which we had data were imperfect proxies. In the case of pasture expansion, head of cattle per square kilometer may be a better indicator of ranching intensification than of geographic expansion. Finally, the process of translation from qualitative themes to spatial, quantitative proxy variables relied on literature reviews and expert judgment and was subject to data availability; this process relies on assumptions and simplifies the nuances of qualitative data (Alcamo, 2008).

Our method, though more time intensive than typical approaches to land‐use‐change modeling, provides a tool for situations in which a higher degree of accuracy and localized nuance are needed for understanding and predicting land use trends. This may be the case where PA managers seek to identify interventions to address the fundamental drivers of deforestation. Combining quantitative modeling with discourse analysis also has the potential to test the explanatory power of narratives around deforestation. Because the stories actors use to explain deforestation shape the proposed solutions, assessing how they relate quantitatively to observed land use trends could help disrupt or shift narratives to better reflect observed changes.

The integration of qualitative discourse analysis methods into land‐use‐change modeling adds precision and nuance to understandings of deforestation in Jamanxim National Forest. By converting themes identified through discourse analysis into spatial, quantitative variables for inclusion in land‐use‐change modeling, we were better able to explain observed deforestation dynamics and may be better able to predict future deforestation hotspots. Despite the challenges and limitations of integrating qualitative and quantitative methodologies and data, our results demonstrate the benefits of this approach for interdisciplinary conservation science.

## Supporting information

Appendix 1: Remote sensing methods
Appendix 2: Discourse analysis methods.
**Table S1**. Documents assessed in the discourse analysis. Full citations for these documents are provided at the end of this appendix section.
**Table S2**. Literature used to identify variables related to deforestation in the Amazon Basin.
**Table S3**. Initial themes identified through a review of deforestation modeling literature and a word count of documents in NVivo. Potential spatial and quantitative proxies are listed for each of the initial themes.
**Table S4**. Emergent themes that were identified during discourse analysis of document subsample, along with potential proxy variables, where applicable.
Appendix 3: Supplementary tables.
**Table S5**. Remote sensing accuracy assessment.
**Table S6**. Transition probability matrix.
