# Supplementary material for: Integration of qualitative and quantitative methods for land‐use‐change modeling in a deforestation frontier
Source: Conserv Biol. 2022 Jun 17;36(6):e13924. doi: 10.1111/cobi.13924 (PMC10084278; doi:10.1111/cobi.13924)
Supplement: Supplementary file 1 — Appendix 1: Remote sensing methods Appendix 2: Discourse analysis methods. Table S1. Documents assessed in the discourse analysis. Full citations for these documents are provided at the end of this appendix section. Table S2. Literature used to identify variables related to deforestation in the Amazon Basin. Table S3. Initial themes identified through a review of deforestation modeling literature and a word count of documents in NVivo. Potential spatial and quantitative proxies are listed for each of the initial themes. Table S4. Emergent themes that were identified during discourse analysis of document subsample, along with potential proxy variables, where applicable. Appendix 3: Supplementary tables. Table S5. Remote sensing accuracy assessment. Table S6. Transition probability matrix. [file COBI-36-0-s001.docx]

# Supplementary Materials

**Contents**

Appendix 1: Remote sensing methods

Appendix 2: Discourse analysis methods

Appendix 3: Supplementary tables

# Appendix 1: Remote sensing methods

Description of the remote sensing methods, to accompany the text in the *Methods* section of the main text.

We performed image compositing and supervised classification in Google Earth Engine and classification validation in R. For cloud-free image compositing, we only chose pixels that had less than 15% cloud and cloud shadow coverage. Our compositing algorithm used the first available cloud-free pixel within our time period (mid-May to mid-October), thus prioritizing earlier months. For ancillary environmental data, we calculated EVI using cloud-free image composites for dry (mid-May to mid-October) and wet (mid-October to mid-May, advancing to subsequent year) seasons. We calculated the difference between wet and dry seasons by subtracting the two values and taking the absolute value of the result. EVI is calculated as 2.5 *((NIR – RED) / (NIR + 6 * RED – 7.5 * BLUE + 1)) (<https://www.usgs.gov/landsat-missions/landsat-enhanced-vegetation-index>).

We performed manual training data collection with Geosurvey (QED, 2019). We classified pasture or croplands as agriculture, recently logged areas and river beds as bare soil, and all buildings as urban. For some training windows in Geosurvey, ongoing land use change was occurring, as indicated by different land covers shown in the various available layers on Geosurvey. Because Geosurvey does not include the exact time that imagery was taken, in these cases we compared Geosurvey imagery to our cloud-free composite. We then chose the Geosurvey layer that best represented land use conditions at the time of our image composite. The lack of time stamps on the Geosurvey imagery is a potential source of error. However, because we were classifying 2018 imagery in 2019, and Geosurvey uses the most up-to-date imagery, this issue should be minimal.

Because our training data points were randomly distributed across the landscape (instead of stratified in land cover types), we had a disproportionately high number of forest points, leading to high accuracy for forest and less than sufficient accuracy for other land classes. Therefore, to improve accuracy, we collected up to 100 extra polygons in non-forest land classes. These extra polygons improved accuracy and were used for the final supervised classification. In the supervised classification, we used all 7 spectral bands, elevation, EVI, and EVI difference as inputs to predict land cover. The random forest model included 100 trees and only used 8 of the input variables for each attempt (Belgiu and Drăguţ, 2016; Breiman, 2001; Liaw and Wiener, 2002).

To test the accuracy of our supervised classification, we used a 10 k-fold cross validation (Cohen, 1960). We then calculated overall accuracy by subtracting from 100 the out-of-bag error from the random forest classification (James et al., 2013; Liaw and Wiener, 2002). Overall accuracy shows what percent of the entire case study area has likely been misclassified (therefore, this accuracy tends to be high due to high accuracy in detecting forest and high forest area). To test classification performance for each land cover individually we calculated user’s and producer’s accuracy measures, evaluating commission and omission errors, respectively.

**Literature cited**

Belgiu, M., Drăguţ, L., 2016. Random forest in remote sensing: A review of applications and future directions. ISPRS J. Photogramm. Remote Sens. 114, 24–31.

Breiman, L., 2001. Random forests. Mach. Learn. 45, 5–32.

Cohen, J., 1960. A coefficient of agreement for nominal scales. Educ. Psychol. Meas. 20, 37–46.

James, G., Witten, D., Hastie, T., Tibshirani, R., 2013. An introduction to statistical learning with applications in R. Springer Texts in Statistics, New York.

Liaw, A., Wiener, M., 2002. Classification and regression by randomForest. R News 2, 18–22.

QED, 2019. Geosurvey [WWW Document]. URL https://geosurvey.qed.ai/about/

.

# Appendix 2: Discourse analysis methods

Description of the discourse analysis methods, to accompany the text in the Methods section of the main text.

**1) Selection of documents**

*Selection criteria:* Documents for the qualitative discourse analysis were identified through a systematic online search based on the following criteria:

a) Documents must mention the name of the designated area OR name of the related province

AND

b) “Deforestation” and/or “Agricultural Development” AND/OR “Agricultural Expansion” (in English or Portuguese)

*Search Protocol:* We used a snowball sampling method to compile policy and management documents. Policy documents were included if documents encompassed the related province (Pará) OR Jamanxim National Forest. In selecting policy documents, we compiled all laws, decrees, institutional regulations, and vetoes of laws that specifically mentioned Jamanxim National Forest. From these documents, the applicable laws, decrees, institutional regulations, vetoes, etc. that were cross-referenced in these documents (as their basis/support) were compiled and included in the sample. We selected gray literature and advocacy documents by identifying all non-governmental and civil society organizations working in the area and locating their online publications. We sampled to the point of saturation, including documents based on relevance and repetition. Our final sample consisted of 61 documents (Table S1).

## **Table S1.** Documents assessed in the discourse analysis. Full citations for these documents are provided at the end of this appendix section.

| Scale | Management | Policy | Gray Literature | Advocacy |
| --- | --- | --- | --- | --- |
| Local (Jamanxim National Forest) | 2 | 4 | 1 | 9 |
| State (Pará) | 0 | 1 | 3 | 7 |
| National (Brazil) | 3 | 7 | 19 | 5 |

*Classification of documents:* We classified documents as management (*e.g.*, park management plans), policy (*e.g.*, laws and decrees related to the protected areas and forest management), gray literature (*e.g.*, reports from government agencies and NGOs), and advocacy (*e.g.*, articles and other documents written by NGOs to promote their campaigns and initiatives or support certain arguments). We also organized the documents by the scale of focus: local (Jamanxim National Forest), Pará (encompassing the protected area), and national.

**2) Establishing Initial Themes**

*Initial Theme Identification:* We established the initial discourse analysis themes through a word count conducted with NVivo 12 software (QSR International Pty Ltd., 2019) for all documents as well as by compiling a list of the traditional variables identified through a review of Amazonian deforestation modeling literature (Table S2). Words or phrases that appeared across the majority of documents were included as initial themes (Table S3).

## **Table S2.** Literature used to identify variables related to deforestation in the Amazon Basin.

| **Literature reviewed** |
| --- |
| Barber, C.P., Cochrane, M.A., Souza, C.M., Laurance, W.F., 2014. Roads, deforestation, and the mitigating effect of protected areas in the Amazon. Biol. Conserv. 177, 203–209. https://doi.org/10.1016/j.biocon.2014.07.004 |
| Costa Roriz, P.A., Yanai, A.M., Fearnside, P.M., 2017. Deforestation and Carbon Loss in Southwest Amazonia: Impact of Brazil’s Revised Forest Code. Environ. Manage. 60, 367–382. |
| Lambin, E.F., Geist, H.J., Lepers, E., 2003. Dynamics of land-use and land-cover change in tropical regions. Annu. Rev. Environ. Resour. 28, 205–241. |
| Molin, P.G., Gergel, S.E., Silvio, B.S.S., 2017. Spatial determinants of Atlantic Forest loss and recovery in Brazil. Landsc. Ecol. 32, 857–870. https://doi.org/10.1007/s10980-017-0490-2 |
| Müller, R., Müller, D., Schierhorn, F., Gerold, G., Pacheco, P., 2012. Proximate causes of deforestation in the Bolivian lowlands: An analysis of spatial dynamics. Reg. Environ. Chang. 12, 445–459. https://doi.org/10.1007/s10113-011-0259-0 |
| Pacheco, P., 2009. Smallholder livelihoods, wealth and deforestation in the Eastern Amazon. Hum. Ecol. 37, 27–41. https://doi.org/10.1007/s10745-009-9220-y |
| Pérez-Vega, A., Mas, J.F., Ligmann-Zielinska, A., 2012. Comparing two approaches to land use/cover change modeling and their implications for the assessment of biodiversity loss in a deciduous tropical forest. Environ. Model. Softw. 29, 11–23. https://doi.org/10.1016/j.envsoft.2011.09.011 |
| Rosa, I.M.D., Purves, D., Souza Jr., C., Ewers, R.M., 2013. Predictive Modelling of Contagious Deforestation in the Brazilian Amazon. PLoS One 8, e77231. https://doi.org/10.1371/journal.pone.0077231 |
| Rosa, I.M.D., Purves, D., Carreiras, J.M.B., Ewers, R.M., 2014b. Modelling land cover change in the Brazilian Amazon: temporal changes in drivers and calibration issues. Reg. Environ. Chang. 15, 123–137. https://doi.org/10.1007/s10113-014-0614-z |
| Schielein, J., Börner, J., 2018. Recent transformations of land-use and land-cover dynamics across different deforestation frontiers in the Brazilian Amazon. Land use policy 76, 81–94. |
| Soares-Filho, B.S., Nepstad, D.C., Curran, L.M., Cerqueira, G.C., Garcia, R.A., Ramos, C.A., Voll, E., McDonald, A., Lefebvre, P., Schlesinger, P., 2006. Modelling conservation in the Amazon basin. Nature 440, 520–523. https://doi.org/10.1038/nature04389 |
| Soares-Filho, B., Rodrigues, H., Follador, M., 2013. A hybrid analytical-heuristic method for calibrating land-use change models. Environ. Model. Softw. 43, 80–87. https://doi.org/10.1016/j.envsoft.2013.01.010 |
| Viteri-Salazar, O., Toledo, L., 2020. The expansion of the agricultural frontier in the northern Amazon region of Ecuador, 2000–2011: Process, causes, and impact. Land use policy 99, 104986. https://doi.org/10.1016/j.landusepol.2020.104986 |

**Table S3.** Initial themes identified through a review of deforestation modeling literature and a word count of documents in NVivo. Potential spatial and quantitative proxies are listed for each of the initial themes.

| **Initial theme** | **Potential proxy variable(s)** |
| --- | --- |
| Physical and economic accessibility | - Distance to roads - Elevation, slope, aspect - Distance to navigable rivers - Distance to cities (markets) - Distance to previously deforested land |
| Population pressure and expansion | - Population density - Population change - Presence of settlements |
| Suitability for agriculture | - Elevation, slope, aspect - Precipitation - Soil moisture |
| Economic activity | - GDP |
| Urbanization | - Urbanization rate |
| Governance | - Administrative jurisdiction (e.g. zoning) |
| Economic activity, Demand for land | - Head of cattle per municipality |
| Poverty | - Poverty rate |

**3) Subsample Coding and Identification of Emergent Themes**

With the initial list of themes, we chose a sub-sample of the total documents (n = 30) with at least one sample from each document classification (*e.g.*, Jamanxim, Local, Management). Two coauthors (MMN and ES) coded these documents in NVivo 12 using the initial themes and creating two broad, umbrella themes entitled “drivers” and “mediators.” When documents discussed deforestation drivers and mediators that were not included in the initial list of themes, we coded these passages by these new themes. Following this initial coding process, we reviewed the “driver” and “solution” codes from all the documents. If a driver or solution was repeated across more than three documents, then we listed it as an emergent theme for the analysis of the full sample of documents (Table S4). Note that not all emergent themes led to a corresponding spatial and quantitative proxy due to a lack of data availability.

**Table S4.** Emergent themes that were identified during discourse analysis of document subsample, along with potential proxy variables, where applicable.

| **Type of emergent theme** | **Emergent theme** | **Potential proxy variable(s)** |
| --- | --- | --- |
| *Drivers* | Ranching | - Head of cattle per km^2^ |
|  | Migration | - Population growth |
|  | Agriculture | - Crop suitability |
|  | Resource extraction | - Illegal logging - Logging - Mining - Unauthorized mining |
|  | Land tenure; settlements; land grabbing | - Agrobusiness expropriation - Indigenous land titling - Land titling - Land grabbing - Pre-existing land claims in the protected area - Smallholder occupation |
|  | Fires | - Distance to fire perimeters - Fire density |
|  | Infrastructure development | - Distance to proposed dams - Distance to proposed railroads - Distance to transmission lines |
|  | Globalization | - Lack of commodity traceability |
|  | Protected area downgrading, downsizing, and degazettement | - Proposed PADDD events - Implemented PADDD events |
| *Mediators* | Sustainable development | - Presence of agroforestry initiatives - Presence of ecotourism |
|  | Indigenous land tenure | - Presence of Indigenous Territories |
|  | Education | - Rate of high school completion |
|  | New protected areas/expanded existing protected areas | - Proposals for new or expanded protected areas - Establishment of new or expanded protected areas |
|  | Economic incentives for forest conservation | - Boycotts - Carbon markets - Payments for ecosystem services - REDD+ |
|  | State governance | - Enforcement - Monitoring - State capacity - Regulatory jurisdiction - Territorial planning - Governance quality |
|  | Non-state governance | - Level of local participation - NGO projects - Environmental education and public outreach |
|  | Government policies | - Agricultural policies - Climate change policy - Forestry policies - Land use policies |

**4) Analysis**

*Discourse Analysis:* We coded the complete sample of 61 documents for both initial and emergent themes in NVivo 12. Following coding, we analyzed each code for relationships between themes, new variables and spatial proxies, and contextual narratives regarding the themes we had identified that could facilitate interpretation of deforestation models.

**5) Translation of themes to spatial proxies**

For each initial and emergent theme identified through the discourse analysis (Tables S3, S4), we identified potential spatial and quantitative or categorical proxies. This translation process allowed us to integrate the qualitative themes of the discourse analysis into the spatial and quantitative land use change models. Our ability to identify appropriate spatial, quantitative proxies was limited by data availability. For some emergent themes, such as “Non-state governance,” we were unable to locate spatial, quantitative proxies. For other emergent themes, such as “Government policies,” there was no spatial variation in the quantitative/categorical proxies, so we did not include these variables in the land use change models, since the value was the same across the entire landscape. We identified potential spatial and quantitative proxy variables through literature review and the best available data (Alcamo, 2008; Mallampalli et al., 2016).

***Discourse analysis reference list***

Abdala, G.C. 2015. The Brazilian Amazon: challenges facing an effective policy to curb

deforestation. Brasilia, Brazil: WWF Living Amazon Initiative & WWF Brazil. pp. 68.

Araújo, E., Barreto, P. 2015. Estratégias e fontes de recursos para proteger as unidades de

conservação da Amazônia. Belém, Pará: Imazon. pp. 40.

Araújo, E., Barreto, P., Martins, H. 2015. Áreas protegidas críticas na Amazônia no período de

2012 a 2014. Belém, Pará: Imazon. pp. 20.

Araújo, E., Barreto, P., Baima, S., Gomes, M. 2016. Quias os planos para proteger as Unidades

de Conservação vulneráveis da Amazônia? Belém, Pará: Imazon. pp. 36.

Araújo, E., Barreto, P., Baima, S., Gomes, M. 2017. Unidades de conservação mais desmatadas da Amazônia Legal 2012-2015. Belém, Pará: Imazon. pp. 92.

Barreto, P. 2009. Como prevenir e punir infrações ambientais em áreas protegidas na

Amazônia? Belém, Pará: Imazon. pp. 52.

Barreto, P., Araújo, E., Brito, B. 2009. A impunidade de crimes ambientais em áreas protegidas

federais na Amazônia. Belém, Pará: Imazon. pp. 56.

Barreto, P., Souza Jr., C. Noguerón, R., Anderson, A., Salomão, R. 2005. Pressão humana na

floresta amazônica brasileira. Belém, Pará: WRI and Imazon. pp. 84.

Cardoso, D., Souza Jr., C. 2018. Sistema de Monitoramento da Exploração Madeireira (Simex): Estado do Pará 2015-2016. Belém, Pará: Imazon. pp. 36.

Coalizão Brasil Clima, Florestas e Agricultura. 2017. Coalizão Brasil Clima, Florestas e

Agricultura to Michel Temer. May 25, 2017.

Corrêa Pinto, I., Vedoveto, M., Veríssimo, A. 2013. Environmental compensation: an

opportunity for consolidating Conservation Units in Pará. Belém, Pará: Imazon. pp. 60.

De Luca, A.C., Develey, P.F., Bencke, G.A., Goerck, J.M. 2009. Áreas importantes para a

conservação das aves no Brasil. Parte II – Amazônia, Cerrado e Pantanal. São Paulo, Brazil: SAVE Brasil. pp. 361.

Government of Brazil. 1998. Lei Nº 9.605. February 12, 1998. Brasilia, Brazil.

Government of Brazil. 2000. Lei Nº 9.985. July 18, 2000. Brasilia, Brazil.

Government of Brazil. 2002. Decreto Nº 4.340. August 22, 2002. Brasilia, Brazil.

Government of Brazil. 2003. Plano de Ação para a Prevenção e o Controle do Desmatamento na

Amazônia Legal. 2^a^ Fase (2009-2011): Rumo ao dematamento illegal zero. November 2009. Brasilia, Brazil. pp. 166.

Government of Brazil. 2006. “Cria a Floresta Nacional do Jamanxim, no Município de Novo

Progresso, no Estado do Pará, e dá outras providências.” February 13, 2006. Brasilia,

Brazil.

Government of Brazil. 2006. Decreto Nº 5.758. April 13, 2006. Brasilia, Brazil.

Government of Brazil. 2006. Lei Nº 11.284. March 2, 2006. Brasilia, Brazil.

Government of Brazil. 2009. Diário Oficial da União, 220. November 18, 2009. Brasilia, Brazil.

p. 59.

Government of Brazil. 2011. Decreto Nº 7.572. September 28, 2011. Brasilia, Brazil.

Government of Brazil. 2012. Lei Nº 12.651. May 25, 2012. Brasilia, Brazil.

Government of Brazil. 2012. Modelo Lógico: Plano de Ação para a Prevenção e o Controle do

Desmatamento na Amazônia Legal (PPCDAm). Brasilia, Brazil. pp. 1.

Government of Brazil. 2013. Plano de Ação para a Prevenção e o Controle do Desmatamento na

Amazônia Legal (PPCDAm). 3^a^ Fase (2012-2015): Pelo uso sustentável e conservação da floresta. Brasilia, Brazil. pp. 171.

Government of Brazil. Bioma Amazonia: Áreas Protegidas: auditoría coordinada. Brasilia,

Brazil: Tribunal de Cuentas de la Unión. pp. 64.

Government of Brazil. 2016. Balanço da 3^a^ fase, 2012-2015, PPCDAm. Brasilia, Brazil. pp. 68.

Government of Brazil. 2016. Planos de Ação para a Prevenção e o Controle do Desmatamento.

Documento base: context e analises. PPCDAm & PPCerrado. Brasilia, Brazil. pp. 85.

Government of Brazil. n.d. Avaliação do Plano de Ação para a Prevenção e o Controle do

Desmatamento na Amazônia Legal, PPCDAm 2007-2010. Brasilia, Brazil. pp. 103.

Government of Brazil. n.d. Plano de Ação para a Prevenção e o Controle do Desmatamento na

Amazônia Legal (PPCDAm): Plano Operativo 2016-2020. Brasilia, Brazil. pp. 60.

Greenpeace Brazil. 2012. Relatório do Greenpeace indica que o desmatamento pode estar na

mesa do brasileiro. <https://www.greenpeace.org/brasil/blog/relatorio-do-greenpeace-indica-que-o-desmatamento-pode-estar-na-mesa-do-brasileiro/>. Accessed May 10, 2019.

Greenpeace Brazil. 2018. Imaginary trees, real destruction. São Paulo, Brazil: Greenpeace

Brazil. pp. 27.

Greenpeace International. 2006. Eating up the Amazon. Amsterdam: Greenpeace International.

pp. 62.

Grupo de Trabalho pelo Desmatamento Zero et al. 2017. Por que o Congresso deve rejeitar

projeto que aumentará o desmatamento da Amazônia? Grupo de Trabalho pelo Desmatamento Zero, Coalizão Pró-UC, Greenpeace Brasil, Instituto Centro de Vida, Imaflora, Instituto de Pesquisa Ambiental da Amazônia, Imazon, Instituto Socioambiental, Rede Pró-UCs, TNC Brasil, & WWF Brasil. pp. 10.

Guetta, M., Oviedo, A. 2019. “Parceria Público-Privada” para o crime ambiental. Instituto

Socioambiental. <https://www.socioambiental.org/pt-br/blog/blog-do-isa/parceria-publico-privada-para-o-crime-ambiental>. Accessed May 10, 2019.

Imazon. 2017. Redução da Flona do Jamanxim: vitória da especulação fundiária?

<https://imazon.org.br/publicacoes/reducao-da-flona-do-jamanxim-vitoria-da-especulacao-fundiaria/>.

Imazon. n.d. Nosso patrimônio amenaçado: Como Unidades de Conservação na Amazônia estão

em risco. Belém, Pará: Imazon. pp. 46.

Instituto Chico Mendes de Conservação da Biodiversidade. 2010. Plano de Manejo: Floresta

Nacional do Jamanxim. Volume II: Planejamento. Brasilia, Brazil.

# International Rivers. 2012. Dilma sacrifices protected areas to accelerate Amazon dam

# construction. <https://archive.internationalrivers.org/resources/dilma-sacrifices-protected-areas-to-accelerate-amazon-dam-construction-3689>. Accessed April 25, 2019.

# International Rivers. n.d. Tapajós basin dams.

# <https://archive.internationalrivers.org/resources/tapaj%C3%B3s-basin-dams-3352>. Accessed April 25, 2019.

# IPAM Amazônia. 2017. Premiando a grilagem na Amazônia: Jamanxim pode ser só o começo.

# <https://ipam.org.br/premiando-a-grilagem-na-amazonia-jamanxim-pode-ser-so-o-comeco/>. Accessed April 24, 2019.

ISA. 2017. Congressistas colocam em risco 1 milhão de hectares de floresta no oeste do Pará.

<https://www.socioambiental.org/pt-br/noticias-socioambientais/congressistas-colocam-em-risco-1-milhao-de-hectares-de-floresta-no-oeste-do-para>. Accessed May 10, 2019.

ISA. 2017. MPF manda parar processo de concessão da Ferrogrão por falta de consulta a povos

indígenas. <https://www.socioambiental.org/pt-br/noticias-socioambientais/mpf-manda-parar-processo-de-concessao-da-ferrograo-por-falta-de-consulta-a-povos-indigenas>. Accessed May 10, 2019.

ISA. 2017. Propostas do governo e do Congresso para Jamanxim também vão beneficiar

mineradoras. Instituto Socioambiental. <https://www.socioambiental.org/pt-br/noticias-socioambientais/propostas-do-governo-e-do-congresso-para-jamanxim-tambem-vao-beneficiar-mineradoras>. Accessed May 10, 2019.

ISA. 2018. Para cumprir acordo internacional, Brasil precisaria reduzir desmatamento em 43%.

Instituto Socioambiental. <https://www.socioambiental.org/pt-br/noticias-socioambientais/para-cumprir-acordo-internacional-brasil-precisaria-reduzir-desmatamento-em-43>. Accessed May 10, 2019.

ISA. 2018. Vitória do meio ambiente: STF veta redução de parques e reservas por Medida

Provisória. <https://www.socioambiental.org/pt-br/noticias-socioambientais/vitoria-do-meio-ambiente-stf-veta-reducao-de-parques-e-reservas-por-medida-provisoria>. Accessed May 10, 2019.

Marinelli, C.E. 2011. De olho nas unidades de conservação : Sistema de Indicadores

Socioambientais para Unidades de Conservação da Amazônia Brasileira. São Paulo, Brazil: Instituto Socioambiental. pp. 12.

Martins, H., Vedoveto, M., Araújo, E., Barreto, P., Baima, S., Souza Jr., C., Veríssimo, A. 2012.

Áreas protegidas críticas na Amazônia legal. Belém, Pará: Imazon. pp. 93.

Ministério do Meio Ambiente & Instituto Chico Mendes de Conservação da Biodiversidade.

2017. Portaria Nº 258. April 17, 2017. Brasilia, Brazil. pp. 4.

Moura, R., Santos, D., Veríssimo, A., Nunes, S., Brito, B., Barreto, P., Martins, H., Celentano,

D. 2017. Desmatamento ZERO no Pará: desafios e oportunidades. Belém, Pará: Imazon. pp. 84.

Pantaleoni Ricardo, H.F., Rolla, A., de Melo Futada, S., & Gomes de Carvalho, F.d. 2019.

Garimpo illegal nas UCs e TIs da Amazônia Brasileira. São Paulo, Brazil: Instituto Socioambiental. pp.16.

Sarney Filho, J. 2016. EM nº00071/2016 MMA. December 14, 2016. Brasilia, Brazil.

Shkrada Resk, S. 2017. Mobilização #Resista visa combater restrocesso ambiental no país. Alta

Floresta and Cuiabá, Brazil: Instituto Centro de Vida.

SOS Amazônia. 2012. Desmatamento avança em unidades de conservaçao.

<http://www.sosamazonia.org.br/>. Accessed April 24, 2019.

Torres, M., Doblas, J., Fernandes Alcaron, D. 2017. “Dono é quem desmata”: Conexões entre grilagem e desmatamento no sudoeste paraense. Altamira, Pará: Instituto Agronômico da Amazônia. pp. 243.

Veríssimo, A., Rolla, A., Vedoveto, M., de Melo Futada, S. 2011. Áreas Protegidas na Amazônia

brasileira: avanços e desafios. Belém and São Paulo: Imazon and Instituto Socioambiental. pp. 87.

WWF Brazil. 2016. A conservation vision for the Tapajos basin. Brasilia, Brazil: WWF Brazil.

pp. 54.

WWF Brazil. 2017. Unidades de Conservação sob Risco: ofensiva contra áreas protegidas

abrange uma área quase do tamanho de Portugal. Brasilia, Brazil: WWF Brazil. pp. 7.

# Xingu Vivo. 2012. Povos do Tapajós apelam ao STF e ao Congresso pela reprovação de MP que

# diminui unidades de conservação no Pará. <https://xinguvivo.org.br/2012/05/15/povos-do-tapajos-apelam-ao-stf-e-ao-congresso-pela-reprovacao-de-mp-que-diminui-unidades-de-conservacao-no-para/>. Accessed April 28, 2019.

# Xingu Vivo. 2012. Organizações socioambientais exigem que Senado não aprove Medida

# Provisória que diminui áreas protegidas na Amazônia. <https://xinguvivo.org.br/2012/05/29/organizacoes-socioambientais-exigem-que-senado-nao-aprove-medida-provisoria-que-diminui-areas-protegidas-na-amazonia/>. Accessed April 28, 2019.

***Literature cited***

Alcamo, J., 2008. The SAS Approach: Combining Qualitative and Quantitative Knowledge in Environmental Scenarios, in: Alcamo, J. (Ed.), Environmental Futures: The Practice of Environmental Scenario Analysis. Elsevier B.V., Amsterdam, pp. 123–150. https://doi.org/10.1016/S1574-101X(08)00406-7

Mallampalli, V.R., Mavrommati, G., Thompson, J., Duveneck, M., Meyer, S., Ligmann-Zielinska, A., Druschke, C.G., Hychka, K., Kenney, M.A., Kok, K., Borsuk, M.E., 2016. Methods for translating narrative scenarios into quantitative assessments of land use change. Environ. Model. Softw. 82, 7–20. https://doi.org/10.1016/j.envsoft.2016.04.011

QSR International Pty Ltd., 2019. NVivo 12.

# Appendix 3: Supplementary tables

## **Table S5.** Remote sensing accuracy assessment.

| Type of accuracy | Ag | Forest | Bare soil | Built-up | Wetland | Water | Mean |
| --- | --- | --- | --- | --- | --- | --- | --- |
| Producer’s | 0.87 | 0.98 | 0.92 | 0.95 | 0.94 | 0.97 | 0.94 |
| User’s | 0.94 | 0.97 | 0.78 | 0.84 | 0.95 | 0.95 | 0.91 |

## **Table S6.** Transition probability matrix.

|  | | 2018 Land cover class | | | | | |
| --- | --- | --- | --- | --- | --- | --- | --- |
|  |  | Ag | Forest | Bare soil | Built-up | Wetland | Water |
| 2008 Land cover class | Ag | 0.698 | 0.181 | 0.107 | 0.005 | 0.009 | 0.000 |
|  | Forest | 0.050 | 0.940 | 0.007 | 0.000 | 0.003 | 0.000 |
|  | Bare soil | 0.680 | 0.139 | 0.165 | 0.014 | 0.002 | 0.000 |
|  | Built-up | 0.296 | 0.044 | 0.163 | 0.496 | 0.000 | 0.000 |
|  | Wetland | 0.057 | 0.065 | 0.000 | 0.000 | 0.877 | 0.000 |
|  | Water | 0.004 | 0.108 | 0.000 | 0.000 | 0.000 | 0.888 |
